# The *Rhipicephalus (Boophilus) microplus Bm86 *gene plays a critical role in the fitness of ticks fed on cattle during acute *Babesia bovis *infection

**DOI:** 10.1186/1756-3305-3-111

**Published:** 2010-11-19

**Authors:** Reginaldo G Bastos, Massaro W Ueti, Donald P Knowles, Glen A Scoles

**Affiliations:** 1 Program in Vector-Borne Diseases, Department of Veterinary Microbiology and Pathology, Washington State University, Pullman, WA 99164-7040, USA; 2 Animal Disease Research Unit, USDA Agricultural Research Service, Pullman, WA 99164-6630, USA

## Abstract

**Background:**

*Rhipicephalus *(*Boophilus*) *microplus *is an economically important tick of cattle involved in the transmission of *Babesia bovis*, the etiological agent of bovine babesiosis. Commercial anti-tick vaccines based on the *R. microplus *Bm86 glycoprotein have shown some effect in controlling tick infestation; however their efficacy as a stand-alone solution for tick control has been questioned. Understanding the role of the *Bm86 *gene product in tick biology is critical to identifying additional methods to utilize Bm86 to reduce *R. microplus *infestation and babesia transmission. Additionally, the role played by *Bm86 *in *R. microplus *fitness during *B. bovis *infection is unknown.

**Results:**

Here we describe in two independent experiments that RNA interference-mediated silencing of *Bm86 *decreased the fitness of *R. microplus *females fed on cattle during acute *B. bovis *infection. Notably, *Bm86 *silencing decreased the number and survival of engorged females, and decreased the weight of egg masses. However, gene silencing had no significant effect on the efficiency of transovarial transmission of *B. bovis *from surviving female ticks to their larval offspring. The results also show that *Bm86 *is expressed, in addition to gut cells, in larvae, nymphs, adult males and ovaries of partially engorged adult *R. microplus *females, and its expression was significantly down-regulated in ovaries of ticks fed on *B. bovis*-infected cattle.

**Conclusion:**

The *R. microplus Bm86 *gene plays a critical role during tick feeding and after repletion during blood digestion in ticks fed on cattle during acute *B. bovis *infection. Therefore, the data indirectly support the rationale for using Bm86-based vaccines, perhaps in combination with acaricides, to control tick infestation particularly in *B. bovis *endemic areas.

## Background

Ticks are blood-feeding arthropods that affect animals and humans both directly by their feeding activity and indirectly by transmitting a wide variety of pathogens ranging from viruses to more complex protozoan parasites. The one-host tick *Rhipicephalus (Boophilus) microplus *is an economically important ectoparasite of cattle involved in the transmission of the apicomplexan protozoan *Babesia bovis*, the etiological agent of bovine babesiosis [1]. Adult females of *R. microplus *acquire *B. bovis *merozoites by ingesting blood from an infected bovine and pass the protozoan transovarially to their larval offspring that can transmit *B. bovis *sporozoites to cattle during subsequent feeding [1-3]. It was recently shown that *R. microplus *females can acquire *B. bovis *from both acute and persistently infected cattle, and efficiently transmit the protozoan transovarially to their larval progeny [4,5].

The control of *R. microplus *relies mostly on the use of acaricides and to a less extent by the use of commercial vaccines based on the Bm86 antigen [6,7]. The presence of chemical residues in food and the recent development of tick populations resistant to acaricides have lead to concerns about the use of chemical acaricides to control *R. microplus *[6]. Bm86-based vaccines have shown some effect on controlling *R. microplus *and other ticks species, and they can also reduce the use of acaricides [7-9]. However, their efficacy as a stand-alone solution for tick control has been a matter of debate [6].

Bm86 is a membrane-bound glycoprotein expressed mainly on the surface of the digestive tract of *R. microplus *females [10,11]. The function of Bm86 has not been completely elucidated; however, it has been shown that this protein contains several epidermal growth factor (EGF)-like domains that may be involved in blood coagulation and cell growth [12]. Bm86 homologues and orthologues from different tick species, such as *Rhipicephalus annulatus*, *Rhipicephalus decoloratus*, *Hyalomma anatolicum anatolicum *and *Rhipicephalus appendiculatus*, have been identified and are potential vaccine candidates for controlling tick infestation [13-15]. In a recent study the silencing of the *R. microplus Bm86 *gene via RNA interference (RNAi) did not significantly affect the fitness of female ticks fed on uninfected cattle [16]. In contrast, Liao and colleagues demonstrated that the silencing of a *Bm86 *homologue from *Haemophysalis longicornis *ticks reduced the weight of engorged females [17]. An improved understanding of the role played by *Bm86 *in *R. microplus*, as well as by the *Bm86 *homologues and orthologues in different tick species, is important for discovering additional methods to use Bm86 in tick control. Additionally, it is also reasonable to investigate the role played by *Bm86 *in *R. microplus *fitness during *B. bovis *infection, considering the presence of cattle concomitantly infested with *R. microplus *and infected with *B. bovis *in endemic areas.

In this study we tested the hypothesis that the silencing of *R. microplus Bm86 *via RNAi affects the fitness of female ticks fed on cattle during acute *B. bovis *infection. The data indicate that, in context with *B. bovis *infection, *Bm86 *plays a critical role during tick feeding and after repletion during blood digestion. Although *Bm86 *silencing significantly reduced the number of tick females that fed to repletion, gene silencing did not affect the efficiency of transovarial transmission of *B. bovis *from surviving tick females to their larval progeny. We also show that *Bm86 *is expressed in larvae, nymphs, adult males, and gut and ovaries of partially engorged females, and its expression significantly reduced in ovaries of ticks fed on *B. bovis*-infected cattle.

## Results

### Expression of the *R. microplus Bm86 *gene

**Figure 1:**
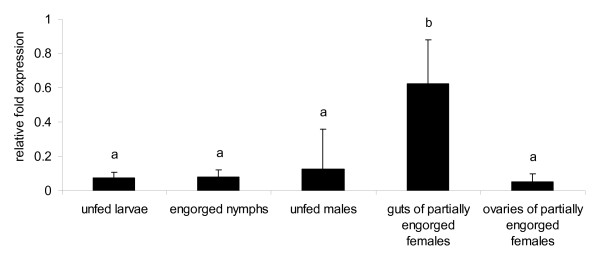
**Expression of the *Bm86 *gene in unfed larvae, engorged nymphs, unfed males, and gut and ovaries of *R. microplus *females**. Six biological replicates of larvae (approximately 100 larvae per sample), engorged nymphs (10 nymphs per sample), unfed males (10 males per sample), and individual ovaries and gut of partially engorged females (at day 5 of feeding) were analyzed by reverse transcriptase quantitative real-time PCR. The transcription level of *Bm86 *was calculate as relative quantity using the delta C*t *comparative method normalized by the total amount of RNA used to generate the cDNA. Different letters (a, b) above the bars indicate significant statistical differences (one-way ANOVA, *F *= 11.456, Tukey post hoc test, *P *< 0.0001).

A reverse transcriptase quantitative real-time PCR (RT-qPCR) was standardized to examine the transcription level of the *R. microplus Bm86 *gene. In a previous study we showed that the *R. microplus *actin, tubulin, *G6PDH*, and *PHGPx*, reference genes commonly used in tick studies, are not stably expressed among tick stages and tick tissues, and therefore they are inadequate for RT-qPCR normalization [18]. Consequently, we decide not to use reference gene(s) and measure the gene transcript level as relative expression normalized by the total amount of RNA used to generate the cDNA. The transcription level was calculated using the following formula: Relative expression _(sample) _= 2 ^[C*t *(control) - C*t *(sample)]^, where the control is a sample with the lowest C*t *value for the gene of interest. The RT-qPCR for *Bm86 *showed efficiency of amplification of 101.9%, with slope of -3.277 (R^2 ^= 0.989) and the range of detection of 10^1 ^to 10^8 ^molecules. Melt curve analyses showed the absence of primer dimers and nonspecific amplification (data not shown). The presence of amplifiable cDNA was examined in all *Bm86 *negative samples by using primers specific for the *R. microplus *actin gene, as previously described [18]. The level of expression of *Bm86 *in different tick tissues and tick stages was investigated in *R. microplus *fed on uninfected calves (*B. bovis*-free calves). Six biological replicates of unfed larvae (approximately 100 larvae per sample), engorged nymphs (10 nymphs per sample), unfed males (10 males per sample), and individual ovaries and gut of partially engorged females (at day 5 of feeding) were analyzed by the *Bm86 *RT-qPCR. Figure 1 shows that *Bm86 *was expressed at different levels in all tick stages and tissues tested in this study. The relative gene expression in the gut samples of partially engorged females (0.622 ± 0.2565) was 4.8, 7.7, 8.4 and 12.5 times higher (*P *< 0.0001) than in the samples of unfed males (0.127 ± 0.2284), engorged nymphs (0.0803 ± 0.0412), unfed larvae (0.073 ± 0.0345) and ovaries of partially engorged females (0.049 ± 0.0485), respectively (Figure 1).

### Effect of *B. bovis *infection on the expression of *Bm86*

**Figure 2:**
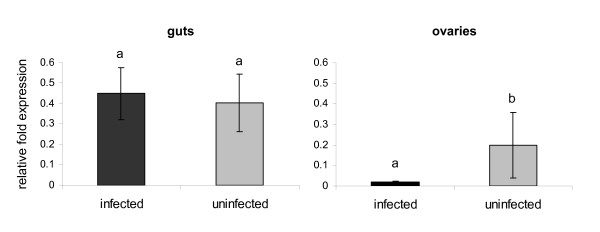
**Effect of *B. bovis *infection on the expression of the *R. microplus Bm86 *gene**. At day 5 of feeding, 6 biological replicates of partially engorged female ticks fed on either *B. bovis*-infected or uninfected cattle were dissected and gene expression was evaluated in individual guts and ovaries by reverse transcriptase quantitative real-time PCR. The transcription level of *Bm86 *was calculate as relative quantity using the delta C*t *comparative method normalized by the total amount of RNA used to generate the cDNA. Different letters (a, b) above the bars indicate significant statistical differences (*t *test, *P *< 0.05).

It has been shown that *B. bovis *infection can decrease the fitness of *R. microplus*, and the severity of the effect is related to the level of parasitemia and tick strain susceptibility [1]. Despite the use of Bm86 in commercial vaccines and the concomitant presence of *R. microplus *infestation and *B. bovis *infection in endemic areas, the effect of the protozoan infection on the expression of *Bm86 *is unknown. Therefore, we examined the transcription level of *Bm86 *in samples of guts and ovaries of *R. microplus *females fed for 5 days on either a *B. bovis*-infected calf or an uninfected calf. Results from 6 biological replicates demonstrated that the relative expression of *Bm86 *in tick guts was not affected by protozoan infection (infected guts: 0.450 ± 0.1274; uninfected guts: 0.405 ± 0.1418; *P *= 0.5608). In contrast, gene expression was significantly lower (*P *< 0.05) in ovaries from ticks fed on *B. bovis*-infected calves (0.017 ± 0.0075) than in ovaries from ticks fed on uninfected calves (0.198 ± 0.1608) (Figure 2).

### Silencing of the *Bm86 *gene

**Figure 3:**
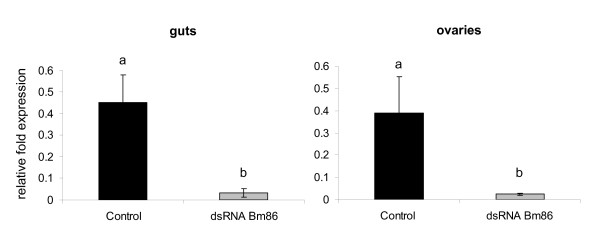
**Level of *Bm86 *transcript in partially engorged *R. microplus *females injected with *Bm86 *dsRNA (grey bar) or buffer control (black bar)**. At day 5 after injection, 6 biological replicates of partially engorged female ticks fed on cattle during acute *B. bovis *infection were dissected and gene expression was evaluated in individual guts and ovaries by reverse transcriptase quantitative real-time PCR. The presented data is representative of ticks from experiment one however, similar levels of gene silenced were obtained in ticks from experiment two. The transcription level of *Bm86 *was calculate as relative quantity using the delta C*t *comparative method normalized by the total amount of RNA used to generate the cDNA. *Bm86 *expression was reduced 92.9% (± 9.3%) and 93.8% (± 2.8%) in gut and ovaries, respectively. Different letters (a, b) above the bars indicate significant statistical differences (*t *test, *P *< 0.05).

To assess the level of gene silencing induced by double stranded RNA (dsRNA), freshly molted unfed females were injected with either dsRNA identical to the *R. microplus Bm86 *gene or buffer control and fed on cattle during acute *B. bovis *infection. At day 5 after injection, 6 biological replicates of individual ovaries and guts were examined by RT-qPCR. In the gut samples, the *Bm86 *gene was silenced 92.9% (± 9.3%) and its relative expression decreased approximately 4 times (*P *= 0.0005) compared to the control samples. In the ovary samples, *Bm86 *was silenced 93.8% (± 2.8%) and its expression decreased more than 6 times (*P *= 0.0003) compared to the control samples (Figure 3).

### Effect of *Bm86 *silencing on tick fitness

**Figure 4:**
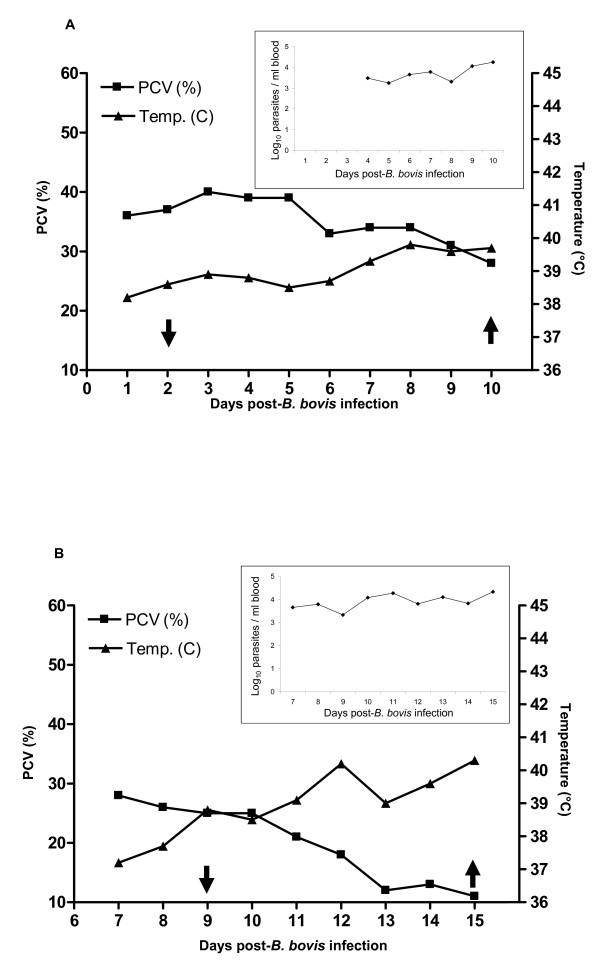
**Results of temperature, packed cell volume (PCV) and *Babesia bovis *parasitemia of calves used to feed the *Bm86 *silenced ticks and control ticks**. Panel A and B show the experimental conditions of experiment one and two, respectively. The upper right chart in the panels A and B show the results of parasitemia of *B. bovis *in peripheral blood of calves of experiment one and two, respectively. Arrows highlight the difference between experiment one and two and indicate the beginning (arrows down) and end (arrows up) of tick feeding period.

The effect of *Bm86 *silencing on the fitness of *R. microplus *females fed on cattle during acute *B. bovis *infection was examined in two independent experiments. The experiments were designed to evaluate the effect of gene silencing on ticks fed on calves undergoing different levels of severity of acute *B. bovis *infection. In experiment one, the dsRNA-injected ticks and control ticks were place to feed on calf #36207 at day 2 after the *B. bovis*-infection whereas in experiment two, the dsRNA-injected ticks and control ticks were placed to feed on calf #1248 at day 8 after the infection (Figure 4A and 4B). In experiment one, during the tick feeding period, the calf temperature ranged from 38.7 to 39.7°C, the calf packed cell volume (PCV) varied from 40 to 28% and the *B. bovis *parasitemia in the calf peripheral blood ranged from 3.4 to 4.3 Log_10 _parasites/ml (Figure 4A). In experiment two, during the tick feeding period, the calf temperature ranged from 37.8 to 40.3°C, the calf PCV varied from 25 to 11% and the *B. bovis *parasitemia in the calf peripheral blood ranged from 3.8 to 4.3 Log_10 _parasites/ml (Figure 4B). At day 15 after the *B. bovis *infection (7 days of tick feeding), the calf #1248 used in experiment two had to be euthanized due to severity of disease caused by the protozoan infection.

**Figure 5:**
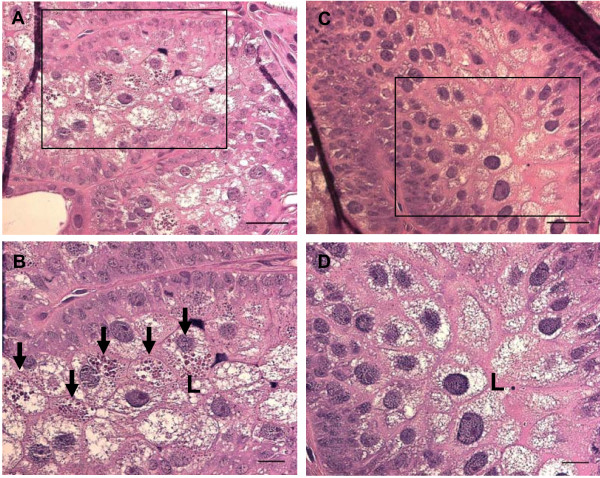
**Histological analysis of *R. microplus *gut sections stained with hematoxylin and eosin and visualized under light microscopy**. A representative gut section of a *Bm86 *silenced tick is shown in panels A and B (magnification of 40× and 63×, respectively) and a representative gut section from a control tick is shown in panels C and D (magnification of 40× and 63×, respectively). The scale bars in the lower right corner of the upper panels represent 50 μm whereas the scale bars in the lower right corner of the lower panels represent 20 μm. The rectangles in the upper panels correspond to the area shown at 63× magnification in the lower panels. The letter "L" indicates the gut lumen and the arrows show the presence of oval-shape corpuscles resembling undigested red blood cells in the apical gut cells of the *Bm86 *silenced tick.

The effect of *Bm86 *silencing on the phenotype of *R. microplus *females was investigated in the two experiments described above. Considering experiment one, a significantly lower (*P *< 0.0001) number of the *Bm86 *silenced females fed to repletion (36 out of 180) compared to the control group (83 out of 180). The weight of the engorged females was not significantly affected by gene silencing despite the tendency of lower weight in the silenced group (319.3 mg ± 81.1) compared to the control group (342.4 mg ± 56.7). The percentage of oviposition in the survivor females was not affected by *Bm86 *silencing; however the weight of egg masses was significantly lower (*P *= 0.0093) in the silenced group (121.0 mg ± 47.9) than in the control group (141.1 mg ± 42.3). The silencing of *Bm86 *did not significantly affect the percentage of hatching and larval survival. The infection rate of *B. bovis *in larval progeny was not affected by gene silencing, despite the significantly lower number of females that fed to repletion in the *Bm86 *silenced group than in the control group. Thirty percent of the larval progeny samples of the *Bm86 *silenced females were infected with *B. bovis *whereas 20% of the larval progeny samples of the control females were infected with the protozoan (Table 1). Additionally, in experiment one, *Bm86 *silenced ticks and control ticks were collected at day 5 of feeding for histological analyses. Hematoxylin and eosin (H&E) staining of tick tissue sections revealed the presence of a number of oval-shape corpuscles resembling undigested red blood cells in the apical gut cells of the *Bm86 *silenced females (Figure 5, panels A and B). The absence of similar structures was noted in gut cells of the control females (Figure 5, panels C and D). Considering experiment two, there was no significant difference in the weight of engorged females between the silenced group and the control group, despite the tendency of lighter females in the silenced group (226.6 mg ± 40.0) than in the control group (233.1 mg ± 41.0) (parametric *t *test, *P *= 0.0479 and non-parametric Mann-Whitney test, *P *= 0.0616). The silencing of *Bm86 *did not significantly affect oviposition, weight of eggs and hatching of the surviving female ticks (Table 1).

**Table 1:** Effect of silencing the *Bm86 *gene on *R. microplus *females fed on cattle during acute *Babesia bovis *infection.

	**Treatments**	**Percentage of engorged females (n)**	**Weight (mg) of engorged females (St.Dev.)**	**% of oviposition (n)**	**Egg mass (mg) (St.Dev.)**	**Percentage of hatching (n)**	**Percentage of larvae survival (n)**	**Infection rate of *B. bovis *in larval progeny (n)**^**e**^
	
Experiment one ^a^	Control females	46.1% (83/180)^b^	342.4 (± 56.7)	98.7% (82/83)	141.1 (± 42.3)	89.9% (74/82)	100% (74/74)	30% (10)
	Silenced females	20% (36/180)^c^	319.3 (± 81.1)	91.3% (33/36)	121.0 (± 47.9)^d^	90.6% (30/33)	100% (30/30)	20% (10)
	
Experiment two ^a^	Control females	ND	233.1 (± 41.0)	87.5% (7/8)	69.0 (± 31.0)	85.7 (6/7)	ND	ND
	Silenced females	ND	226.6 (± 40.0)	100% (8/8)	70.0 (± 47.0)	87.5 (7/8)	ND	ND

^a ^Experimental conditions of experiment one and two are described in Figure 4A and 4B, respectively.

^b ^A total of 200 ticks were injected with *Bm86 *dsRNA however, 20 partially engorged females from each group from both experiments were collected for gene expression and histological analyses, and 180 females were used for fitness evaluation.

^c ^Chi-squared test (*P *< 0.01)

^d ^*t *test (*P *< 0.01)

^e ^Number of larvae progeny samples (containing approximately 100 larvae per sample)

ND - not determined

**Figure 6:**
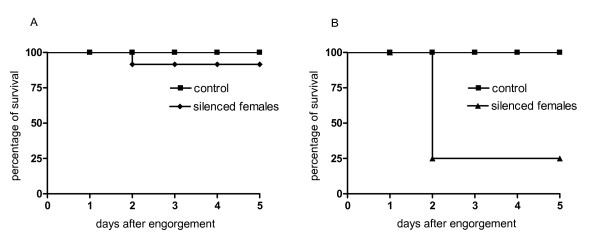
**Kaplan-Meier curves showing the percentage of survival of *Bm86 *silenced and control *R. microplus *engorged females after feeding to repletion on cattle during acute *B. bovis *infection**. Panels A and B show the percentage of survival of female ticks of the experiment one and two, respectively. In experiment one, 100% (n = 83) of the replete females of the control group survived the evaluation period whereas 91.6% (33 out of 36) of the replete females survived in the silenced group. In the experiment two, 100% (n = 8) of the replete females of the control group survived the evaluation period whereas only 27.5% (8 out of 29) of the replete females survived in the silenced group. Chi-squared test showed significant differences (*P *< 0.05) between the control and silenced groups in both experiments.

Interestingly, both experiments demonstrated that the silencing of *Bm86 *significantly decreased (*P <*0.05) the percent of survival of replete females in the first 5 days after dropping from the *B. bovis*-infected calves (Figure 6). In experiment one, 100% (n = 83) of the replete females of the control group survived the evaluation period whereas 91.6% (33 out of 36) of the replete females survived in the silenced group (Figure 6A). In experiment two, 100% (n = 8) of the replete females of the control group survived the evaluation period whereas only 27.5% (8 out of 29) of the replete females survived in the silenced group (Figure 6B). Notably, in both experiments, the replete *Bm86 *silenced females died at day 2 after dropping from the *B. bovis*-infected calves and they presented a dark coloration suggesting leakage of the gut content into the tick hemolymph.

## Discussion

It has been shown that Bm86-based vaccines affect the fitness *of R. microplus *by decreasing the number, weight and fecundity of engorging females [7,9]. It has also been reported that anti-Bm86 sera affect the endocytotic activity of *R. microplus *gut cells [10]. In the present study we took one step further and showed that the silencing of the *Bm86 *gene decreases the fitness of *R. microplus *ticks fed on *B. bovis*-infected cattle. Evidence from two independent experiments demonstrated that *Bm86 *plays a role during the feeding period and blood digestion in *R. microplus *females fed on cattle acutely infected with *B. bovis*. Gene silencing significantly decreased the number and percentage of survival of engorged females, and decreased the weight of egg masses. We also show that *Bm86 *is expressed in larvae, nymphs and adults of *R. microplus *and its expression was significantly down-regulated in ovaries of ticks fed on cattle during acute *B. bovis *infection.

It was recently shown that silencing of *Bm86 *had no statistical effect on the fitness of *R. microplus *females fed on uninfected cattle, despite the tendency of lower tick weight and egg mass weight in the silenced group than in the control group [16]. In contrast, it has been demonstrated that the silencing of a *Bm86 *homologue of *H. longicornis *ticks significantly decreased the weight of engorged females [17]. Our data show that the silencing of the *R. microplus Bm86 *gene significantly decreased tick fitness in the presence of acute *B. bovis *infection. The *B. bovis*-infected calves used in this study showed evidence of acute infection during the tick feeding period. Clinical indications of acute infection included a drop in PCV, fever and detection of parasites in peripheral blood by PCR. As shown in Figure 4B, there was a profound drop in PCV of the calf used in experiment two and the animal had to be euthanized 15 days after the *B. bovis *infection (7 days of tick feeding). As a result, clinical signs and parasitemia may have affected the tick fitness; however the overall biological impact cannot be solely attributed to *B. bovis *infection, considering the significant biological differences between the *Bm86 *silenced group and control group.

Two independent experiments were performed to investigate the effect of *Bm86 *silencing on *R. microplus *fitness in context with *B. bovis *infection. Although comparisons between experiments were not intended, some aspects of both experiments should be addressed en bloc. In experiment one, the weight of egg mass was significantly decreased by gene silencing; however, it was not significantly affected in experiment two. Additionally, there was a pronounced difference in survival of the replete females between experiment one and experiment two. These discrepancies may be explained by the different number of female ticks evaluated in each experiment, and by the fact that in experiment two the effect of gene silencing was investigated only in the most rapidly engorging cohort of females fed on a calf experiencing severe acute *B. bovis *infection. Notably, the silencing of *Bm86 *significantly decreased the survival of engorged *R. microplus *females and this effect was pronounced in experiment two where the PCV dropped from 25 to 11% during the tick feeding period. Therefore, it is reasonable to hypothesize that the effect of gene silencing on the mortality of engorged females may have been exacerbated by low PCV values. However, caution should be exercised when interpreting data from experiment two considering that it was untimely terminated due to the severity of the *B. bovis *infection and consequently only a small number of engorged *R. microplus *females were evaluated. Experiment one showed no significant differences in the infection rate of *B. bovis *in larval progeny from the *Bm86*-silenced group and the control group, demonstrating that gene silencing did not affect transovarial transmission of the parasite. It has been demonstrated that *B. bovis*-infected *R. microplus *larval progeny are very efficient in transmitting the parasite [4,5]. The effect of *Bm86 *silencing on the ability of larval progeny to transmit *B. bovis *was not tested in this study; however, considering the published data and the infection rates of the larval progeny in the present study, there was no rationale to expect that larval progeny would not transmit the parasite in subsequent feedings.

Amino acid analyses have revealed that Bm86 contains several EGF-like domains [12]. Proteins containing EGF-like regions fall into two general categories: those involved in blood coagulation and complement cascade, and those associated with the regulation of cell growth [19]. It has been proposed that Bm86 resembles the latter group, which is characterized by multiple EGF repeats, transmembrane regions and location on the extracellular surface [12]. It has been also argued that Bm86 could act as a cell membrane-bound ligand transmitting positional or cell-type information to adjacent cells in a manner similar to the Drosophila Notch protein [12]. It has been reported that anti-Bm86 sera affect the endocytotic activity of tick gut cells, suggesting that this protein is somehow involved in the intracellular process of blood digestion [10]. Here we show that silencing of the *Bm86 *gene in context with *B. bovis *infection decreased the number of engorging females and caused an increase in mortality of replete females. Additionally, histological analyses revealed the presence of structures resembling undigested red blood cells in gut cells of *Bm86 *silenced ticks. Despite the low number of ticks evaluated in the histological analyses, the data suggest that silencing of *Bm86 *caused some level of impairment in digesting the blood meal. It was beyond the scope of this study to perform a full functional analysis of the *Bm86 *gene, but the data demonstrate that *Bm86 *plays a role in blood digestion during engorgement and survival after repletion. Considering the histological results and the significantly higher mortality of replete females in the silenced group than in the control group, we hypothesize that silencing of *Bm86 *compromised the ability of the guts to maintain and digest a blood meal from cattle acutely infected with *B. bovis*.

It has been demonstrated that Bm86 protein is present mainly on the surface of the digestive tract of *R. microplus *females [10,11]. However, information about gene expression and/or presence of the protein in other tick tissues and in different tick stages is scarce. This study shows that the *Bm86 *gene is expressed at different levels in larvae, nymphs and adult *R. microplus*, confirming previous observation [16]. Although gene expression is not necessarily an indication of protein expression, it is reasonable to expect the presence of the Bm86 protein in other tick stages than adult females and in other tick tissues than gut, and this could be an important factor for the efficacy of Bm86 vaccines as a direct binding site for anti-Bm86 antibodies. The relevance of this result needs to be investigated in context with the efficiency of Bm86-based vaccines to control one-host and multiple-host tick species.

It has been shown that *Babesia spp *infection can affect tick fitness and the severity of these effects is related to the degree of parasitemia [1]. In fact, it was recently demonstrated that *B. bovis *infection changes the protein expression profile of *R. microplus *females [20,21]. Our results reinforce the published data and show that the expression of *Bm86 *decreased significantly in ovaries of *R. microplus *fed on cattle during *B. bovis *infection. The biological relevance of this data needs to be further addressed in context with protozoa infection and the use of Bm86-based vaccines.

Off-target effects caused by dsRNA have been described in numerous species [22-24] and cannot be entirely ruled out as the cause of the results observed in this study. The *R. microplus *genome sequence is not currently available [25], consequently alignment analyses are restricted to DNA and cDNA tick sequences listed in the GenBank and Gene Index Project databases http://compbio.dfci.harvard.edu. The 403-bp sequence used to synthesize the dsRNA does not have significant identity to any known sequence other than with the *R. microplus Bm86 *gene. Additionally, we demonstrate that the *R. microplus Bm86 *gene was significantly silenced by the injection of the 403-bp dsRNA. Collectively, these aspects support the phenotype data present in this study and represent the best possible strategy to make solid scientific observation regarding the biological effect of the silencing of *Bm86 *in *R. microplus *in context with *B. bovis *infection.

In conclusion, the results show that *Bm86 *plays a critical role during tick feeding and after repletion during blood digestion in ticks fed on cattle during acute *B. bovis *infection. The data indirectly support the rationale for using Bm86-based vaccines, perhaps in combination with acaricides, to control tick infestation. This strategy may be particularly relevant for controlling ticks in *B. bovis *endemic areas, considering the assumption that *Bm86 *plays a more critical role in the fitness of *R. microplus *fed on *B. bovis*-infected cattle than in ticks fed on uninfected animals [16]. Interestingly, the efficiency of transovarial transmission of *B. bovis *from surviving tick females to their larval progeny was not affected by silencing *Bm86*, despite the lower number of females that fed to repletion in the silenced group than in the control group. This study also demonstrated that *Bm86 *is expressed in larvae, nymphs, males, and gut and ovaries of female *R. microplus *ticks and its expression was down-regulated in ovaries by *B. bovis *infection. Additional investigations are required to elucidate the function of *Bm86*, and *Bm86 *homologues and orthologues, and its interaction with protozoa infection. Efforts should also be concentrated in discovering novel tick antigens and chemical acaricides that, in combination with Bm86-based vaccine, could be used in the development of an efficient program to control tick infestation and protozoa transmission.

## Materials and methods

### Cattle, *B. bovis *and *R. microplus*

Four Holstein calves (#1243, #1248, #1235 and #36207) 3-4 months of age were used in this study. The animals had no previous exposure to ticks and were tested negative for *B. bovis *by cELISA and nested PCR [4,26]. The calves #1243 and #1235 were maintained uninfected throughout the study whereas calves #1248 and #36207 were experimentally infected with approximately 1.4 × 10^8 ^*B. bovis*-infected erythrocytes (T2Bo strain) [27]. The infected calves were monitored daily for the presence of *B. bovis *in peripheral blood and clinical signs of babesiosis. Parasitemia of *B. bovis *in peripheral blood was examined by qPCR to amplify the single copy *msa-1 *gene as previously described [18]. All calves were maintained throughout the experiment in accordance to protocols approved by the University of Idaho Institutional Animal Care and Use Committee. To obtain unfed adult ticks, approximately 40,000 larvae from 2.0 g of eggs of *R. microplus *La Minita strain [28] were placed under a cloth patch on the uninfected calves. On day 13-14, engorged nymphs were manually removed and held in an incubator at 25°C to molt to adults. After 2-3 days of incubation, freshly molted unfed adult females and males were sorted out and used for evaluation of gene expression.

### Synthesis of double stranded RNA

To obtain *Bm86 *dsRNA, 1 μg of total RNA from guts of *R. microplus *females was used for cDNA synthesis using the Superscript Vilo cDNA Synthesis Kit (Invitrogen, USA) following the manufacturer's protocol. The sequence of the *R. microplus Bm86 *Mozambique strain (GenBank accession number EU191620) was used to design primers to amplify by PCR a fragment of 403 base pairs from nucleotide 71 to 473 (5'cagaggatgatttcgtgtgc3' and 5'ccctgacaacaacgagaatccctt3'). The 403-bp amplicon was cloned into pCR^®^II-TOPO^® ^(Invitrogen) and used as template for the dsRNA synthesis using the MEGAscript^® ^Kit (Ambion, USA) according to the manufacturer's protocol. The *Bm86 *dsRNA was checked by electrophoresis on agarose gel, quantified by spectrophotometry and kept at -20°C until used for tick injection.

### Injection of *R. microplus *with double stranded RNA

The effect of *Bm86 *silencing on tick fitness was evaluated in two independent experiments and the procedures for tick injection were similarly performed on both experiments. Individual females were injected with 1 μl of *Bm86 *dsRNA (approximately 1 × 10^11 ^molecules dissolved in 0.1 mM EDTA buffer) or buffer control (0.1 mM EDTA buffer) through the coxal membrane at the base of the 4th leg on the right ventral side, as previously described [18]. Injections were accomplished using a 10 μl syringe with a 33 gauge needle (Hamilton, Bonaduz, Switzerland) and the microprocessor controlled UMP3 injection pump apparatus (World Precision Instruments, Berlin, Germany). In experiment one, 200 dsRNA-injected females, plus an equal number of males, and 200 buffer-injected females, plus an equal number of males, were placed under individual stockinet sleeves glued to the side of the *B. bovis*-infected calf #36207 (2 days after the experimental *B. bovis *infection). In experiment two, 200 dsRNA-injected females, plus an equal number of males, and 200 buffer-injected females, plus an equal number of males, were placed under individual stockinet sleeves glued to the side of the *B. bovis*-infected calf #1248 (8 days after the experimental *B. bovis *infection). At day 5 after injection, 20 partially engorged females from each group from both experiments were collected for gene expression and histological analyses, and 180 females were used for fitness evaluation.

### Evaluation of *R. microplus *fitness

Individual stockinet sleeves of each group from both experiments described above were checked daily for the presence of engorged female ticks. Fully-engorged females were collected, weighed and put in individual wells in 24-well plates at 26°C for oviposition. At day 14 after the beginning of oviposition, egg masses laid by each individual female were weighed and put in individual vials to evaluate hatching. Hatching was evaluated at 30 days after the egg masses were weighed and hatching positive was defined as the presence of any larvae from eggs of an individual female. The larval progeny was maintained in individual vials at 26°C for 45 days and the larval survival was determined as the presence of any live larvae in larval progeny from individual females. At this point, the larval progeny of females injected with either dsRNA or buffer was also tested for the presence of *B. bovis *by nested PCR, as previously described [18].

### Transcription level of the *Bm86 *gene

The pattern of expression and silencing of *Bm86 *was investigated by RT-qPCR in 6 partially engorged female ticks from experiment one and in 6 biological replicates of partially engorged female ticks from experiment two. Tick samples were collected in RNAlater (Ambion) and total RNA was extracted using the RNAqueous^® ^Kit (Ambion). The samples were treated with DNase I (Invitrogen) and the RNA concentration determined by Qubit^® ^Flurometer (Invitrogen). Two hundred ng of total RNA from each sample were used for cDNA synthesis using the Superscript Vilo cDNA Synthesis Kit (Invitrogen) according to the manufacturer's protocol. Technical replicates were performed to evaluate enzymatic variations during the synthesis of cDNA in a given RNA sample. The sequence of the *R. microplus Bm86 *Mozambique strain was used to design RT-qPCR primers (5' gattctcgttgttgtcag 3' and 5' gcaagcatttttacactg 3') to amplify a fragment of 117 base pairs from nucleotide 383 to 499. The RT-qPCR were performed in a CFX96™ Real-Time PCR Detection System (Bio-Rad, Hercules, CA, USA) using the Express SYBR^® ^GreenER™ Supermix Kit (Invitrogen). The cycling conditions consisted of a Uracil-DNA Glycosylase inactivation step of 50°C for 30 sec, initial denaturation of 95°C for 2 min followed by 40 cycles of 95°C denaturation for 15 sec and annealing/extension of 60°C for 45 sec. Reactions were performed in duplicate in 20 μl using 200 nM of each primer and 2 μl of a 1/20 dilution of cDNA as template. An inter-run calibrator was included to assess inter-run variations. The CFX Manager™ Software (Bio-Rad) was used to analyze the RT-qPCR data. Efficiency of amplification and melt curve analyses were performed to evaluate analytical sensitivity and specificity of the RT-qPCR.

### Histological analysis

At day 5 after injection, 3 partially engorged *R. microplus *females from either the *Bm86 *silenced group or control group were collected, fixed in 10% formaldehyde and embedded in paraffin. Five serial sections (4-μm) from each tick were deparaffinized and stained with H&E for light microscopy evaluation. The H&E stained tick sections were viewed and photographed using the Axio Imager Software (version 4.8.1) (Carl Zeiss Microimaging, Thornwood, NY, USA).

### Statistical analyses

The relative gene expression and weights of engorged females and egg masses were compared by ANOVA, *t*-test and Tukey's test (GraphPad Instat^®^, version 3.06, GraphPad Software, Inc., San Diego, CA, USA). The percentages of engorged females, oviposition, hatching and larvae survival were compared by Chi-squared (GraphPad Instat^®^). Tick survival was compared by Kaplan-Meier curves (GraphPad Prism^®^, version 4.00, GraphPad Software, Inc.) and percentage of survival of engorged females was compared by Chi-squared test (GraphPad Instat^®^).

## Competing interests

The authors declare that they have no competing interests.

## Authors' contributions

RGB designed and performed the experiment, and wrote the first draft of the manuscript. MWU designed and performed the experimental procedures involving ticks. DPK designed, supervised and acquired funding for the experiment. GAS designed and performed the experiment. All authors read and approved the final version of the manuscript.
